# Black hole in WONderland: endoscopic management of walled-off necrosis complicated by giant colonic fistula

**DOI:** 10.1055/a-2860-0907

**Published:** 2026-05-12

**Authors:** Rintaro Fukuda, Tomotaka Saito, Shuichi Tange, Tsuyoshi Hamada, Naminatsu Takahara, Yousuke Nakai, Mitsuhiro Fujishiro

**Affiliations:** 1 Department of Endoscopy and Endoscopic Surgery 26782 The University of Tokyo HospitalTokyoJapan; 2 Department of Gastroenterology 317970 The University of Tokyo Graduate School of Medicine School of Medicine Department of GastroenterologyTokyoJapan; 3 Department of Hepato-Biliary-Pancreatic Medicine, Cancer Institute Hospital 13609 Japanese Foundation For Cancer ResearchTokyoJapan; 4 Department of Internal Medicine, Institute of Gastroenterology 13131 Tokyo Women's Medical UniversityTokyoJapan

An endoscopic step-up approach is the standard treatment for walled-off necrosis (WON); however, in patients with multiple organ failure, endoscopic or surgical intervention may be unsafe 1 2 3 . Herein, we report a multidisciplinary step-up strategy for extensive necrotizing pancreatitis complicated by pseudoaneurysm rupture and a giant colonic fistula after transarterial embolization (TAE), with percutaneous stabilization followed by endoscopic intervention and ultimately surgical fecal diversion to achieve definitive control ( Video 1 ).

Endoscopic management of walled-off necrosis complicated by a giant colonic fistula using a multidisciplinary step-up strategy.Video 1

**Fig. 1:**
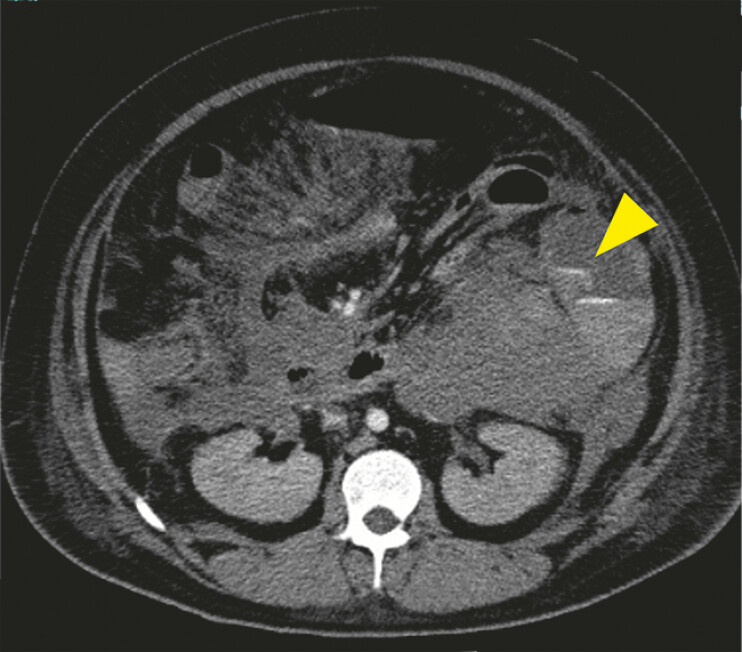
Contrast-enhanced computed tomography demonstrated a ruptured left colic artery pseudoaneurysm. Active extravasation (arrowhead) from the aneurysm is visible, with retroperitoneal hematoma.

**Fig. 2:**
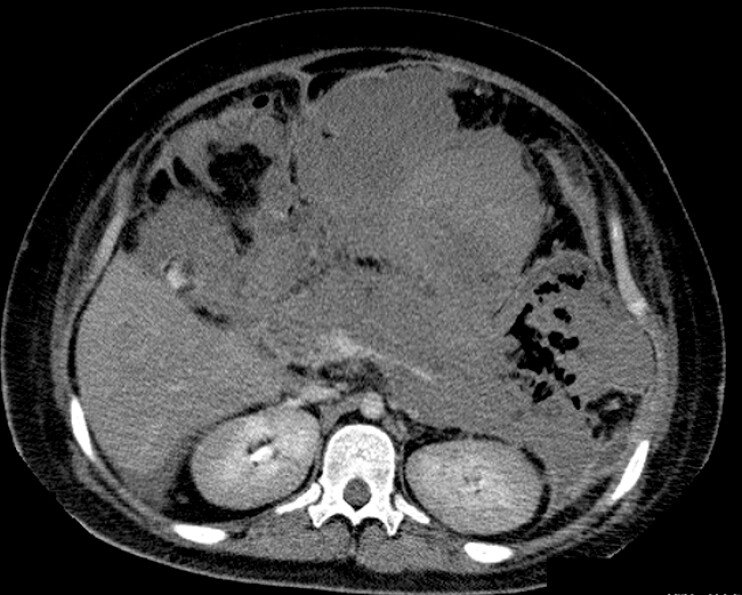
Computed tomography on day 42 of pancreatitis showed extensive walled-off necrosis. The lesion extended caudally into the pelvic cavity prior to intervention.

**Fig. 3:**
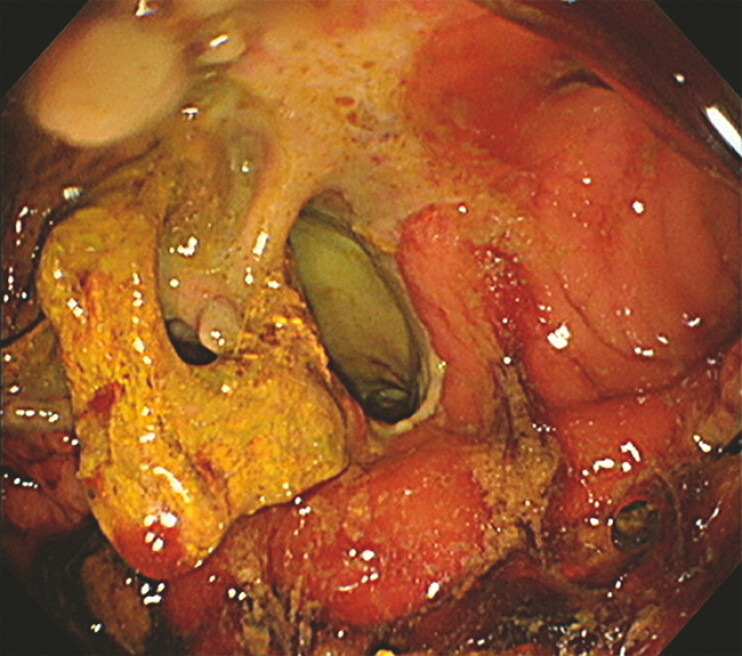
Endoscopy revealed a fistula between the walled-off necrosis and the descending colon. During percutaneous necrosectomy, extensive sloughing of the colonic wall was observed, suggesting ischemic injury due to prior embolization.

**Fig. 4:**
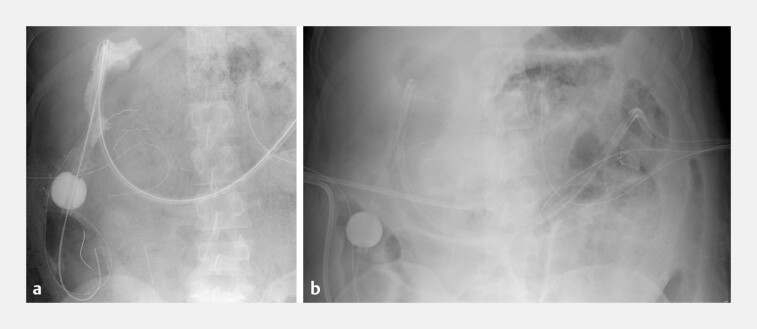
Fluoroscopic and radiographic images of ileus tube placement for fecal diversion. **a** During the procedure, an endoscope was inserted percutaneously through the drainage tract and advanced into the ascending colon under fluoroscopic guidance. **b** A follow-up abdominal radiograph confirmed the appropriate placement of the long tube for fecal diversion.

**Fig. 5:**
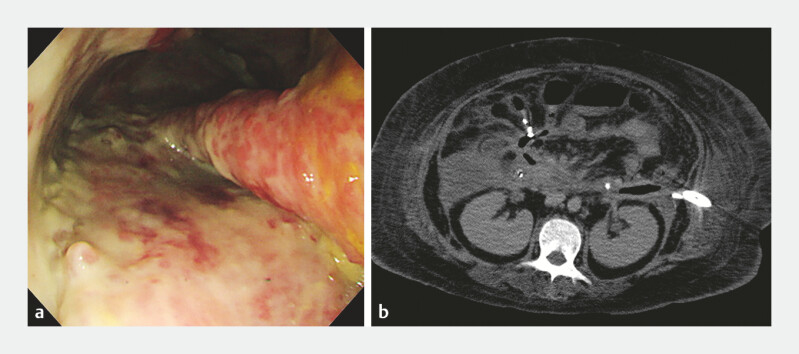
Final images following the completion of treatment. **a** Endoscopy revealed the complete resolution of necrotic tissue. **b** Computed tomography showed a significant reduction in the size of the walled-off necrosis cavity.

A 20-year-old man was transferred to our institution with severe acute pancreatitis of unknown etiology with multiple organ failure requiring intensive care unit admission. On day 22 after the onset of pancreatitis, he suffered cardiopulmonary arrest due to rupture of a left colic artery pseudoaneurysm ( Fig. 1 ), and emergency TAE was performed. Despite improvement in pancreatitis, infected WON progressed ( Fig. 2 ). Endoscopic access was challenging because of respiratory instability, leading to percutaneous drainage followed by step-up necrosectomy 4 . During necrosectomy, a giant fistula between the WON and the descending colon was identified, possibly related to severe inflammation and ischemia after TAE ( Fig. 3 ). Fecal contamination caused refractory infection, but surgical fecal diversion was initially deemed unfeasible due to the patient’s critical condition and severe intra-abdominal inflammation. Therefore, a long ileus tube was percutaneously inserted through the drainage tract to divert fecal flow ( Fig. 4 ), helping stabilize the patient and allowing endoscopic ultrasound-guided drainage with lumen-apposing metal stents and transluminal necrosectomy. Although contamination persisted, repeated necrosectomy achieved partial infection control and reduced intra-abdominal inflammation, allowing surgical fecal diversion. A surgical fecal diversion was performed, resulting in definitive infection control and further clinical improvement ( Fig. 5 ). Necrosectomy progressed, and the necrotic material was cleared after 14 sessions.

This case highlights a multidisciplinary step-up strategy for unstable colonic fistula-associated WON, in which temporary percutaneous ileus tube diversion served as a salvage bridge to definitive surgical management.

Endoscopy_UCTN_Code_TTT_1AS_2AJ
